# Synergistic Effects of Apigenin and Paclitaxel on Apoptosis of Cancer Cells

**DOI:** 10.1371/journal.pone.0029169

**Published:** 2011-12-21

**Authors:** Yimiao Xu, Yinqiang Xin, Ying Diao, Changyan Lu, Jin Fu, Lan Luo, Zhimin Yin

**Affiliations:** 1 Jiangsu Province Key Laboratory for Molecular and Medicine Biotechnology, College of Life Science, Nanjing Normal University, Nanjing, Jiangsu, People's Republic of China; 2 State Key Laboratory of Pharmaceutical Biotechnology, School of Life Sciences, Nanjing University, Nanjing, People's Republic of China; Wayne State University School of Medicine, United States of America

## Abstract

**Background:**

It was well known that the clinical use of chemotherapeutic drugs is restricted by severe adverse reactions and drug resistances. Thus it is necessary to figure out a strategy to increase the specific anti-tumor efficiency of chemotherapeutic drugs. Apigenin, a kind of flavonoids, has been reported to possess anticancer activities with very low cytotoxicity to normal tissue.

**Methodology/Principal Findings:**

Our results from cell viability assay, western-blots and TdT-mediated dUTP-biotin nick end labeling (TUNEL) assay demonstrated the synergistic pro-apoptotic effects of a low dose of apigenin and paclitaxel in human cancer cell lines. To analyze the underlying mechanism, we examined reactive oxygen species (ROS) staining after cells were treated with a combination of apigenin and paclitaxel, or each of them alone. Data from flow-cytometry showed that superoxides but not reduction of peroxides accumulated in HeLa cells treated with apigenin or a combination of apigenin and paclitaxel. Apigenin and paclitaxel-induced HeLa cell apoptosis was related to the level of ROS in cells. We further evaluated activity and protein level of superoxide dismutase (SOD). Apigenin significantly inhibited SOD activity but did not alter the SOD protein level suggesting that apigenin promoted ROS accumulation through suppressing enzyme activity of SOD. Addition of Zn^2+^, Cu^2+^ and Mn^2+^ to cell lysates inhibited apigenin's effects on SOD activity. At the same time, data from caspase-2 over-expression and knocked-down experiments demonstrated that caspase-2 participated in apigenin and paclitaxel-induced HeLa cell apoptosis.

**Conclusions/Significance:**

Taken together, our study demonstrated that apigenin can sensitize cancer cells to paclitaxel induced apoptosis through suppressing SOD activity, which then led to accumulation of ROS and cleavage of caspase-2, suggesting that the combined use of apigenin and paclitaxel was an effective way to decrease the dose of paclitaxel taken.

## Introduction

Chemotherapy is one of the most widely employed treatments for cancer. However many chemotherapeutic drugs can produce unpleasant side effects,especially when taken in high doses. One of the chemotherapeutic drugs, paclitaxel, a mitotic inhibitor, may lead to hypersensitivity reactions [1], neutropenia [2], neurotoxicity [3], cardiac rhythm disorder [4] and other miscellaneous toxic effects [5], which seriously worsens the quality of life of cancer patients and results in dosage reduction and discontinuation of treatment. It is therefore important to decrease the adverse side effects of chemotherapeutic agents in clinical treatment of cancer. In addition, drug resistance in clinical therapy often interferes with the efficiency of chemotherapeutic agents.

Reactive oxygen species (ROS) including superoxide radical, hydrogen peroxide (H_2_O_2_), hydroxyl radical, nitric oxide, and various nitric oxide-derived reactive nitro species (RNS) are formed as natural byproducts of normal metabolism of oxygen in human cells and tissues. Because of their highly reactive character, they tend to become involved in unwanted reactions that cause damage to cells and ultimately lead to diseases. Cancer cells exhibit increased glycolysis in combination with a reduced rate of respiration and these alterations in metabolism have been shown to be associated with enhanced oxidative stress [6]–[8]. A high cell redox status could stimulate tumor formation through creating an enhanced cell-proliferative environment, inducing DNA damage, and turning off tumor suppression functions [9], [10]. Tumor growth and migration could be inhibited *in vitro* by alteration of the environment around tumor cells to a more reducing one. In opposition to this, a high cell redox state would also support increased apoptosis, which would inhibit tumor formation. Thus, in cancer cells, the high redox state could enhance their tolerance to environmental stresses and chemotherapeutic drugs. Tumor cells expressed a higher level of MnSOD indicate a poor prognosis [11], [12]. It has been shown that ROS have potential ability to process caspase-2 [13], [14] which is an initiator caspase led to mitochondrial membrane permeabilization [15] and is also an important member in apoptosis signal amplification loop [16]. Besides, previous studies in caspase-2 knocked-out mice have shown that caspase-2 activation was related with ROS accumulation [17]. Reduced apoptosis rate was also detected in *caspase-2^−/−^* oocytes [18].

Apigenin (4′, 5, 7-trihydroxyflavone) is widely contained in many fruits and vegetables. Recently, it was reported that apigenin had a potential anti-tumor effects on several human cancer cell lines with low cytotoxicity and no mutagenic activity. [19]–[21]. Apigenin could enhance the intracellular accumulation of ROS and had the pro-oxidant potential [22], [23] and decrease SOD activity in lung cancer cells [24].

In the present work, we demonstrated that apigenin could sensitize cancer cells to paclitaxel induced apoptosis through suppressing SOD activity and leading to accumulation of ROS and cleavage of caspase-2, suggesting the combined use of apigenin and paclitaxel was effective for cancer therapy.

## Materials and Methods

### Cell culture and transfection

Human cervical epithelial carcinoma cell line HeLa, human lung epithelial carcinoma cell line A549, human negroid hepatocyte carcinoma cell line Hep3B, and human embryonic kidney 293A (HEK293A) cells obtained from Institute of Biochemistry and Cell Biology, Chinese Academy of Sciences (Shanghai, P.R. China), were maintained in Dulbecco's modified Eagle's medium (Invitrogen) containing 10% fetal calf serum (Hyclone) and antibiotics (100 µg/ml penicillin and 100 µg/ml streptomycin) with 5% CO_2_ at 37°C. Transient transfection was performed with a modified calcium phosphate method or by using the Lipofectamine 2000 reagent (Invitrogen) according to the manufacturer's instructions. In all cases, the total amount of DNA was normalized by the empty control plasmids.

### Antibodies, regents and DNA constructs

Mouse monoclonal antibody against Flag-tag was purchased from Sigma. Rabbit polyclonal antibodies against poly ADP-ribose polymerase (PARP), caspase-3 and β–actin were obtained from Cell Signaling Technology. Mouse polyclonal antibody against caspase-2 came from Santa Cruz Biotechnology. Rabbit polyclonal antibodies against cleaved caspase-3 came from Bioworld Technology, Inc. Mouse monoclonal antibody against SOD1 and rabbit polyclonal antibodies against SOD2, Endo G and AIF were obtained from Abcam. Caspase-2 inhibitor benzyloxycarbonyl-Val-Asp (OMe)-Val-Ala-Asp(OMe)-fluorom ethylketone (z-VDVAD-fmk) was obtained from Calbiochem. ROS detection reagents (CM-H_2_DCFDA and dihydroethidium (DHE)) and apoptotic detecting regent (FITC-Annexin V and propidium iodide buffer) were from Molecular Probes™ (Invirogen). Dimethyl sulfoxide was from Amresco. Apigenin, paclitaxel and DETC were purchased from Sigma.

pcDNA3.1-flag-caspase2 (WT) and pRNAU6-caspase2 were constructed by using standard techniques. pcDNA3.1 was digested with XhoI and KpnI. The primers (sense:TTTTCTCGAGACCATGGACTACAAGGACGACGATGATAAGGCGGCGCCGAGCGCGGGGT, anti-sense: ATATGGTACCTCATGTGGGAGGGTGTCCTG) were designed to generate pcDNA3.1-flag-caspase2 (WT). *Caspase-2* (NM_032982.2) gene was synthesized by Invitrogen. pRNAU6 was digested with Bam HI and Hind III, and the annealed targeting oligonucleotides ACAGCTGTTGTTGAGCGAA for *caspase-2* was ligated into the vector [25].

### Cell viability and TUNEL assay

To evaluate paclitaxel-induced cytotoxicity, Colorimetric Cytotoxic 96 nonradioactive cytotoxicity assay (Promega) was performed according to the manufacturer's protocol. The cell viability was detected by measuring endogenous lactate dehydrogenase quantitatively.

TdT-mediated dUTP-biotin nick end labeling (TUNEL) assay was performed in HeLa cells by using Guava® TUNEL Kit as previously described [26]. The cells were detected on a Guava EasyCyte™ System, and data were analyzed by using Guava TUNEL Software (Guava Technologies, Hayward, CA, USA). All assays were performed for three replicates.

### Annexin V/PI assay

Annexin V/PI assay, by using annexin V/PI double staining of unfixed cells, distinguishes between early apoptotic cells and late apoptotic/necrotic cells. Both floated and attached cells were suspended in 500 µl binding buffer (10 mM HEPES, 140 mM NaCl, 2.5 mM CaCl_2_, 0.1% BSA). Annexin V-FITC (5 µL) and PI (5 µL) was then added into each sample. After 15 min incubation in the dark, the flow cytometric analysis was carried out using the Guava EasyCyte™ System. For each sample 5000 cells were analyzed. Data were analyzed by using Guava TUNEL Software (Guava Technologies, Hayward, CA, USA).

### ROS detection

HeLa cells were seeded in 6-well plates and after 24 h, cells were incubated with ROS specific dyes, dihydroethidium (DHE, 5 µM) or CM-H_2_DCFDA (5 µM), for 30 minutes. Cells were subsequently treated with apigenin (15 µM), paclitaxel (4 nM) or both of them for another 30 minutes. ROS were detected by using a Guava EasyCyte™ and analyzed with the Guava Express Pro Software (Guava Technologies, Hayward, CA, USA). DHE was detected under an emission within 580–583 nm (PM1) and CM-H_2_DCFDA was detected under an emission of 525 nm (PM3).

### Isolation of mitochondrion and Measurement of mitochondrial membrane potential (MMP)

Intact mitochondrion was separated from cytosolic component of HeLa cells for further protein analysis, using Mitochondria Isolation Kit from Thermo-Pierce Dounce homogenization and differential centrifugation were performed according to the manufacturer's protocol.

Mitochondrial membrane potential (MMP) of HeLa cells was measured by using the Guava EasyCyte™ MitoPotential Kit (Guava Technologies, Hayward, CA, USA) as per the manufacturer's instructions. Flourescence-based dye 5, 5′, 6, 6′-tetrachloro-1, 1 ′, 3, 3 ′-tetrethyl benzimidalyl carbocyanine iodide (JC-1) was used to evaluate MMP changes. The cell impermeant dye 7-AAD was used to simultaneously monitor cell membrane permeability changes. Stained cells were analyzed on a Guava EasyCyte™ System.

### Assay of superoxide dismutase activity

The enzyme activity of SOD in HeLa cells was measured by using commercial kits according to manufacturer's protocol. In this assay, the tetrazolium salt was utilized for detection of superoxide radicals generated by xanthine oxidase and hypoxanthine. To measure MnSOD activity, cell lysates were centrifuged at 10,000×g to separate MnSOD from CuZnSOD, and CuZnSOD was inhibited in the presence of potassium cyanide.

### RT-PCR analysis

Total RNA was extracted by using High Pure RNA Isolation Kit (Roche) according to the protocol described by the manufacturer. RT-PCR was carried out using the M-MLV reverse transcriptase (Invitrogen) with indicated primers (*caspase-2*, sense: TTTTCTCGAGACCATGGACTACAAGGACGACGATGATAAGGCGGCGCCGAGCGCGGGGT, anti-sense: ATATGGTACCTCATGTGGGAGGGTGTCCTG; GAPDH, sense: CATATGGGGAAGGTGAAGGTCGGAGTC, anti-sense: GAATTCTTACTCCTTGGAGGCCATGTGG). PCR was performed at Tm of 58°C for 30 cycles in reaction mixture of 25 mL individually of each protein. PCR products were then resolved on 1% agarose gels and stained with ethidium bromide. The house keeping gene GAPDH was utilized as a control.

### Western-blot analysis

Cell lysates were centrifuged (15,000 g) at 4°C for 15 min. The Western blot analysis was performed as previously described [27]. The antibody–antigen complexes were visualized by the LI-COR Odyssey Infrared Imaging System according to the manufacturer's instruction using IRDye800 flurophore-conjugated antibody (LI-COR Biosciences, Lincoln, NE).

### Determination of Synergism

The drug combination was determined by Chou-Talalay method. The CI was calculated by the Chou-Talalay equation, which takes into account both the potency (IC_50_ or D_m_) and the sharp of the dose-effect curve (m value) [28], [29]. The classic isobolgram (CI = 1) is given by:
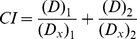
(A)


In the denominators, (D_x_)_1_ and (D_x_)_2_ are the concentrations for D_1_ (apigenin) and D_2_ (paclitaxel) used alone that give x% inhibition, whereas in the numerators, (D)_1_ and (D)_2_ are the doses of apigenin and paclitaxel used in combination that isoeffectively inhibit x%. CI<1, CI = 1, and CI>1 suggest synergism, additive and antagonism, respectively.

From the median-effect equation of Chou and Chou et al. [29], [30], the (D_x_)_1_ and (D_x_)_2_ can be readily calculated.
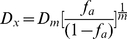
(B)


Here D_m_ is the median-effect dose which obtained from the anti-log of the X-intercept of the median-effect plot, X-log (D) versus Y = log [f_a_/(1−f_a_)] or D_m_ = 10^−(Y−intercept)/m^, and m is the slope of the median-effect plot. Automated calculation of m, D_m_, D_x_, and CI values are also allowed with computer software.

### Statistical analysis

Data was represented as mean±SD. Student's t-test was applied to analyze the statistical significance of variance pair-wise comparison. In all analyses, P<0.05 was considered as statistically significant.

## Results

### Combination treatment of apigenin with paclitaxel significantly enhances cytotoxicity to human cancer cells

**Figure 1:**
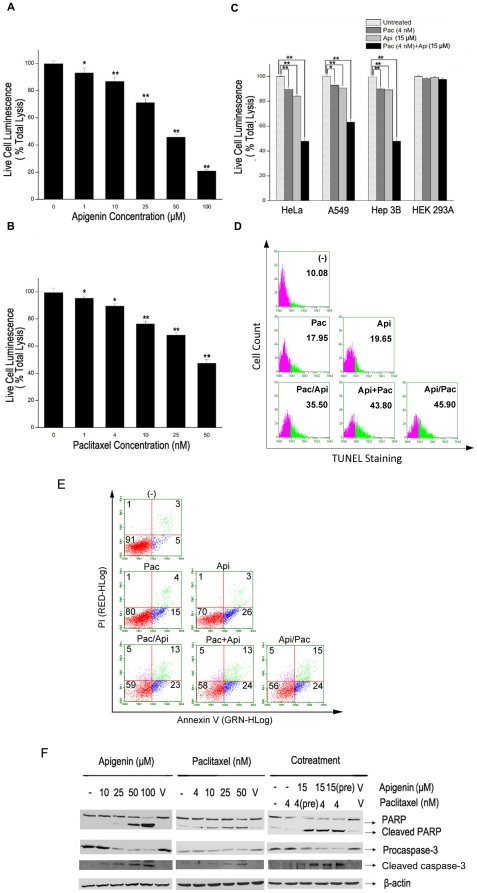
Apigenin and paclitaxel enhance cytotoxicity to cancer cells compared with paclitaxel. A, HeLa cells were treated with increased concentrations of apigenin (0–100 µM) for 24 hours. Cell vitality was measured. The experiment was independently repeated for three times and data were shown as mean ± SD. B, HeLa cells were treated with increased concentrations of paclitaxel (0–50 nM) for 24 hours. Cell vitality was detected and analyzed as described in A. C, HeLa, A549, Hep3B and HEK293A cells were subjected to treatment with apigenin (15 µM) combination with paclitaxel (4 nM) for 24 hours. Cell vitality was detected as described in A. D, HeLa cells were treated with apigenin or paclitaxel respectively, besides, in other groups, apigenin was added to HeLa cells 2 hours before or after paclitaxel treatment, or apigenin and paclitaxel were added to HeLa cells at the same time. TUNEL staining was performed and detected with a Guava® TUNEL Kit. Numbers depicted the percentage of TUNEL-positive cells. E, Cells were either left untreated or treated as previously described in D. At the indicated time, cells were stained with annexin V/PI dye. Analyses were conducted on 5,000 cells in each trail. F, HeLa cells were treated with increased concentrations of apigenin or paclitaxel respectively, or both of them for 24 hours. Then the cell lysates were subjected to Western blot to determine protein level of caspase-3 and PARP. V represents vehicle (dimethyl sulfoxide). *p<0.05 compared with untreated group; **p<0.01 compared with untreated group.

Since toxicity of paclitaxel is coupled to its antitumor activity and apigenin has been reported to have antitumor activity with low toxicity, we first observed the co-effects of comcination paclitaxel with apigenin. HeLa cells were treated with various doses of apigenin, paclitaxel or both of them and then the cell viabilities were detected. As shown in Fig. 1A and B, both apigenin and paclitaxel dose dependently induced cytotoxicity with approximately 29% reduction of cell viability induced by apigenin at the dose of 25 µM (Fig. 1A) and 24% reduction induced by paclitaxel at the dose of 10 nM (Fig. 1B) respectively. When we treated HeLa cells with 15 µM apigenin and 4 nM paclitaxel, over 50% decrease of cell viability was detected, nevertheless, less than 20% decrease of cell viability was observed in cells treated with apigenin or paclitaxel respectively (Fig. 1C). We carried out Chou-Talalay calculation to confirm the synergistic effects of apigenin and paclitaxel on HeLa cells. The combination index (CI) was 0.3918±0.0436 at the dose of 15 µM apigenin and 4 nM paclitaxel indicating synergistic effects of apigenin and paclitaxel. These results indicated a significantly increased cytotoxicity when apigenin and paclitaxel were administrated to HeLa cells simultaneously. Also shown in Fig. 1C, combination of apigenin with paclitaxel induced the similar results in the cancer cells other than Hela cells including Hep3B and A549 cells. However, 15 µM apigenin, 4 nM paclitaxel or combination of them did not result in cytotoxicity to HEK293A cells respectively (Fig. 1C). These data suggested there were synergistic effects of apigenin and paclitaxel for specifically killing cancer cells

It has been reported that both paclitaxel [31], [32] and apigenin [21] can induce cell apoptosis. We next verify whether combination use of apigenin and paclitaxel could induce more acute apoptosis in HeLa cells. Compared to those groups treated with apigenin or paclitaxel individually, apigenin and paclitaxel co-treated groups showed more TUNEL positive staining cells. The rates of TUNEL positive staining cells were notably increased from 17.95% in paclitacel treated group and 19.65% of apigenin treated group to around 40% in the groups treated by combination of them (Fig. 1D). To quantify apoptosis, FACS analysis was carried out after staining cells with FITC-annexin-V plus propidium iodide (PI). Data shown in Fig. 1E indicated that the number of surviving cells decreased gradually after co-treatment with apigenin and paclitaxel and only less than 60% were survived 24 hours after apigenin and paclitaxel treatment.

Since activation of caspases is an important reason for apoptosis, we further detected the cleavages of caspase-3 and PARP using western blot analysis. As shown in Fig. 1F, both the cleavages of caspase-3 and PARP were significantly enhanced by apigenin/paclitaxel co-treatment compared with apigenin or paclitaxel treatment alone. These results confirmed that apigenin/paclitaxel co-treatment induced a significant apoptotic death of HeLa cells suggesting that combination use of apigenin and paclitaxel would produce a better anti-tumor effect than use of apigenin or paclitaxel alone.

### ROS produced by apigenin is essential for apigenin/paclitaxel-induced HeLa cell apoptosis

**Figure 2:**
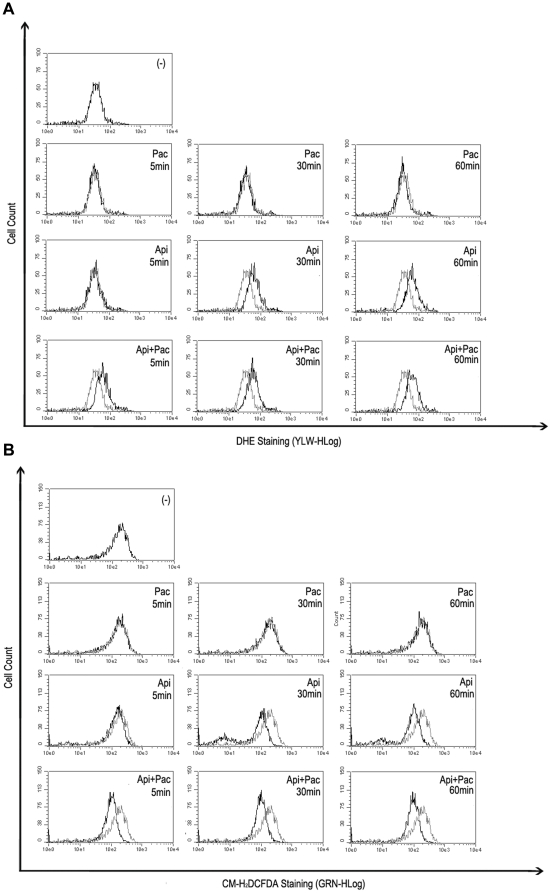
Apigenin induces ROS accumulation in HeLa cells. A, HeLa cells were treated with apigenin (15 µM) alone or together with paclitaxel (4 nM), for indicated periods. DHE (5 µM) was added into cell cultures 30 minutes before the treatment. DHE staining in cells were detected and analyzed by Guava EasyCyte™ System. DHE staining in untreated cells were used as the negative control. B, CM-H_2_DCFDA (5 µM) staining was performed and analyzed as described in A.

Meanwhile flavonoids including apigenin were widely recognized as the antioxidants, their pro-oxidant properties have also been reported [33]. We speculated the anticancer effects of combination apigenin/paclitaxel might be related with production of ROS in HeLa cells induced by apigenin. Therefore we observed the levels in HeLa cells treated with with apigenin, paclitaxel or both of them. Two ROS-specific dyes, DHE and CM-H_2_DCFDA displayed superoxide species and H_2_O_2_ in target cells respectively [24]. CM-H_2_DCFDA is mainly oxidized by hydrogen peroxides (H_2_O_2_) and hydroxyl radical, DHE is oxidized by superoxide anions (O_2_^−^). HeLa cells were stained with DHE and CM-H_2_DCFDA followed by flow cytometry. As shown in Fig. 2, DHE-specific ROS increased (Fig. 2A) whereas CM-H_2_DCFDA-specific ROS reduced (Fig. 2B) in HeLa cells treated with apigenin within the 30 minutes. No change was detected in the cells treated with paclitaxel alone. A similar trend and a more significant change in ROS' status were observed in those cells treated with combination of apigenin and paclitaxel compared with cells treated with apigenin alone (Fig. 2). DHE staining observed in these experiments resulted from the oxidation by superoxide species suggesting that apigenin increased superoxide specie related ROS in HeLa cells.

**Figure 3:**
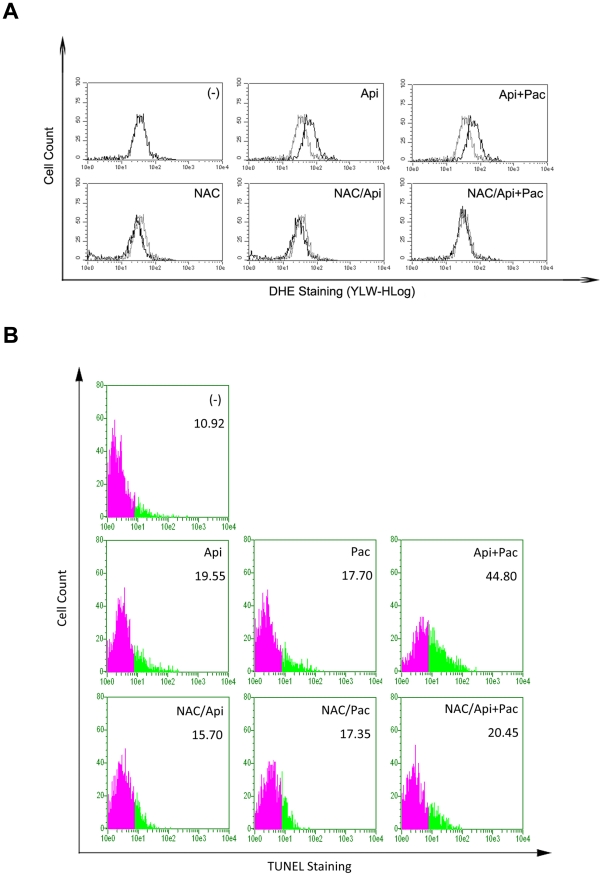
ROS accumulation is required for apigenin/paclitaxel-triggered apoptosis A, HeLa cells were treated with ROS scavenger NAC (100 µM) for 2 hours or not, then stained with DHE (5 µM), followed by the treatment with apigenin (15 µM) and paclitaxel (4 nM) for 60 minutes. Cells were then collected and detected by Guava EasyCyte™ System as described in Fig. 2A. B, HeLa cells were treated with NAC (100 µM) for 2 hours and then incubated with apigenin (15 µM) and paclitaxel (4 nM) for 24 hours. Cell apoptosis was measured by TUNEL assay as described in Fig. 1.

Next we evaluated whether apigenin/paclitaxel-induced apoptosis was dependent on ROS accumulation. N-acetyl-L-cysteine (NAC), a well approved ROS scavenger, has been used to antagonize the effect of ROS. Pretreatment of HeLa cells with 100 µM NAC effectively suppressed the generation of superoxide species (Fig. 3A) and significantly inhibited the cell apoptosis induced by apigenin and paclitaxel co-treatment (Fig. 3B). The above data suggested that ROS were essential for apigenin/paclitaxel-induced HeLa cell apoptosis.

### Apigenin induces ROS accumulation through suppressing the activity of SOD

**Figure 4:**
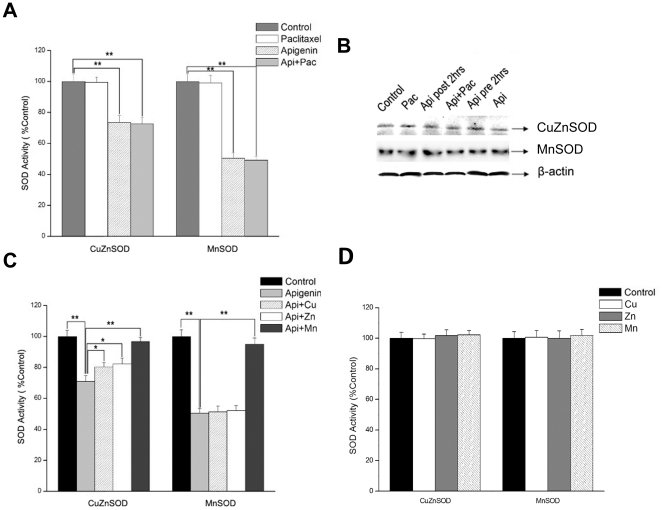
Suppression of SOD activity by apigenin is associated with metal ions in HeLa cells. A, HeLa cells were treated with apigenin (15 µM), paclitaxel (4 nM), or both of them for 30 minutes. SOD activity was measured with Cayman SOD detection kit. The experiment was independently repeated for three times and data were shown with mean±S.D. *p<0.05 compared with control group; **p<0.01 compared with control group. B, HeLa cells were treated with apigenin (15 µM) and paclitaxel (4 nM) for 30 minutes. Cell lysates were subjected to Western-blot Protein to determine levels of SOD1 and SOD2. C, HeLa cells were treated with apigenin (15 µM) for 30 minutes and lysed by sonication. Cell lysates were then incubated with CuCl_2_ (8 µM), ZnCl_2_ (8 µM) and MnCl_2_ (8 µM) for 30 minutes on ice. The SOD activity were measured and analyzed as described in A. D, HeLa cells were lysed by sonication and cell lysates were then incubated with above mentioned metal ions for 30 minutes on ice. SOD activity was measured as mentioned above. *p<0.05 compared with apigenin-treated group; **p<0.01 compared with apigenin-treated group.

We observed that after HeLa cells were treated with apigenin, the abundance of superoxide species increased whereas H_2_O_2_ decreased. Considering SOD was a cellular enzyme which can convert superoxide to H_2_O_2_, we speculated that apigenin might reduce the activity of SOD and consequently led to the accumulation of superoxide species. We then investigated the effect of apigenin on protein level as well as the enzyme activity of SOD. A significant suppression of both CuZnSOD (SOD1) and MnSOD (SOD2) activity was observed in HeLa cells within 30 minutes after apigenin or apigenin/paclitaxel but not paclitaxel treatment (Fig. 4A). However, there was no apparent change observed in the protein level of CuZnSOD and MnSOD after apigenin administration (Fig. 4B). Accordingly, it was evidential that apigenin suppressed SOD activity and thus induced the accumulation of ROS.

As above results showed that apigenin suppressed SOD activity, and it had been reported that apigenin could form stable complexation with metal ions *in vitro* [34], we thus determined if apigenin could form complexation with metal ions and suppress SOD activity through preventing SOD from assemble with its cofactors. HeLa cells were treated with 15 µM apigenin for 30 minutes and the lysates of cells were incubated with 8 µM of ZnCl_2_ (aq), CuCl_2_ (aq) or MnCl_2_ (aq) respectively on ice for another 30 minutes. The activity of SOD was measured using the Cayman Superoxide Dismutase Assay Kit as described in Material and Methods. As expected, apigenin significantly suppressed the activities of both CuZnSOD and MnSOD, but the activity CuZnSOD increased after Cu^2+^, Zn^2+^, and Mn^2+^ exposure and the activity of MnSOD also increased obviously after Mn^2+^ exposure (Fig. 4C). Additionally, no significant change of SOD activity was found when cell lysates were directly exposed to Cu^2+^, Zn^2+^ or Mn^2+^ without apigenin treatment (Fig. 4D), indicating that these metal ions did not enhance basal SOD activity.These results suggested that apigenin suppressed SOD activity probably through forming a stable complexation with those metals in cells [34], and apigenin might have higher binding affinity to Mn^2+^.

### SOD activity reduction is critical in apigenin/paclitaxel-induced apoptosis

**Figure 5:**
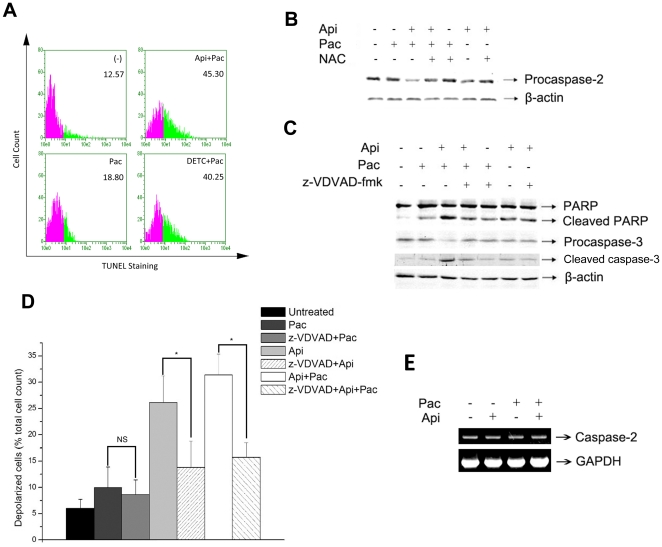
Apigenin-induced ROS are required for cleavage of caspase-2, MMP and cell apoptosis. A, HeLa cells were incubated with apigenin (15 µM)/paclitaxel (4 nM), or paclitaxel (4 nM) together with or without 1 mM DETC (an inhibitor of SOD) for 24 hours and then cells were subjected to TUNEL assay as described previously. B, HeLa cells were pretreated with NAC (100 µM) for 2 hours, followed by the treatment with apigenin (15 µM) and paclitaxel (4 nM) for 24 hours. Cleavage of caspase-2 was detected by Western-blot analysis with the control of β-actin. C, HeLa cells were pretreated with z-VDVAD-fmk (25 µM), a specific inhibitor of caspase-2, and then were treated with apigenin (15 µM) and paclitaxel (4 nM) for 24 hours. Protein levels of cleaved PARP and procaspase-3 were detected by Western-blot analysis. D, HeLa cells were pretreated with z-VDVAD-fmk (25 µM) for 30 minutes and then were treated with apigenin (15 µM) and paclitaxel (4 nM) for 8 hours. Cells were then harvested and mitochondrial membrane potential was detected using a Guava EasyCyte™ MitoPotential Kit and analyzed by Guava EasyCyte™ System. MMP was showed as the count of depolarized cells. The experiment was independently repeated for three times and data were shown as mean±SD. **p<0.01 compared with untreated group. E, HeLa cells were incubated with apigenin (15 µM) and paclitaxel (4 nM) for 24 hours, and then cells were subjected to RT-PCR analysis. GAPDH mRNA was used as house keeper gene.

Apigenin inhibited the activity of SOD and combination of apigenin/paclitaxel was more effective in inducing apoptosis of cancer cells than each of them alone. In order to confirm that apigenin-induced decrease of activity of SOD was critical in the apoptosis triggered by apigenin/paclitaxel co-treatment, we observed paclitaxel-induced apoptosis after blocking SOD enzyme activity. DETC (diethylthiocarbamate), a well approved inhibitor of both CuZnSOD and MnSOD was applied in our present study [35]. As similar as apigenin, DETC suppressed the activity of SOD and enhanced paclitaxel induced apoptosis as well (Fig. 5A). These data strongly suggested that reduction of SOD activity by apigenin is critical in the enhancement of paclitaxel-induced apoptosis.

### Activation of caspase-2 is important for apigenin/paclitaxel-triggered HeLa cell apoptosis

Mitochondrial pathway plays an important role in ROS triggered apoptosis [36], [37] and caspase-2 has been considered as the apical caspase in mitochondrial death signal amplification loop [38], [39]. ROS-induced caspase-2 cleavage and feedback amplification of the apoptotic signal have also been investigated [40]. To examine the effect of apigenin on mitochondrial pathway of apoptosis, caspase-2 precursor cleavage was detected in apigenin-treated HeLa cells by western-blot analysis. Co-treatment of HeLa cells with apigenin and paclitaxel showed very low level of caspase-2 precursor. When HeLa cells were pretreated with NAC, the caspase-2 precursor level recovered (Fig. 5B). The caspase-2 inhibitor z-VDVAD-fmk significantly reduced the cleavage of caspase-3 and PARP in HeLa cells treated with apigenin combined with paclitaxel (Fig. 5C). MMP may be a causal event in precipitating apoptosis. The data in Fig. 5D indicated that apigenin but not paclitaxel induced an increase of depolarization of MMP suggesting the importance of apigenin in apigenin/paclitaxel induced cancer cell apoptosis. As expected, z-VDVAD-fmk inhibited the increase of MMP in apigenin and apigenin/paclitaxel treated groups (Fig. 5D). Fig. 5E showed that treatment of apigenin, paclitaxel or apigenin/paclitaxel did not alter the mRNA level of caspase-2 indicating apigenin/paclitaxel co-treatment only increased activation of caspase-2.

**Figure 6:**
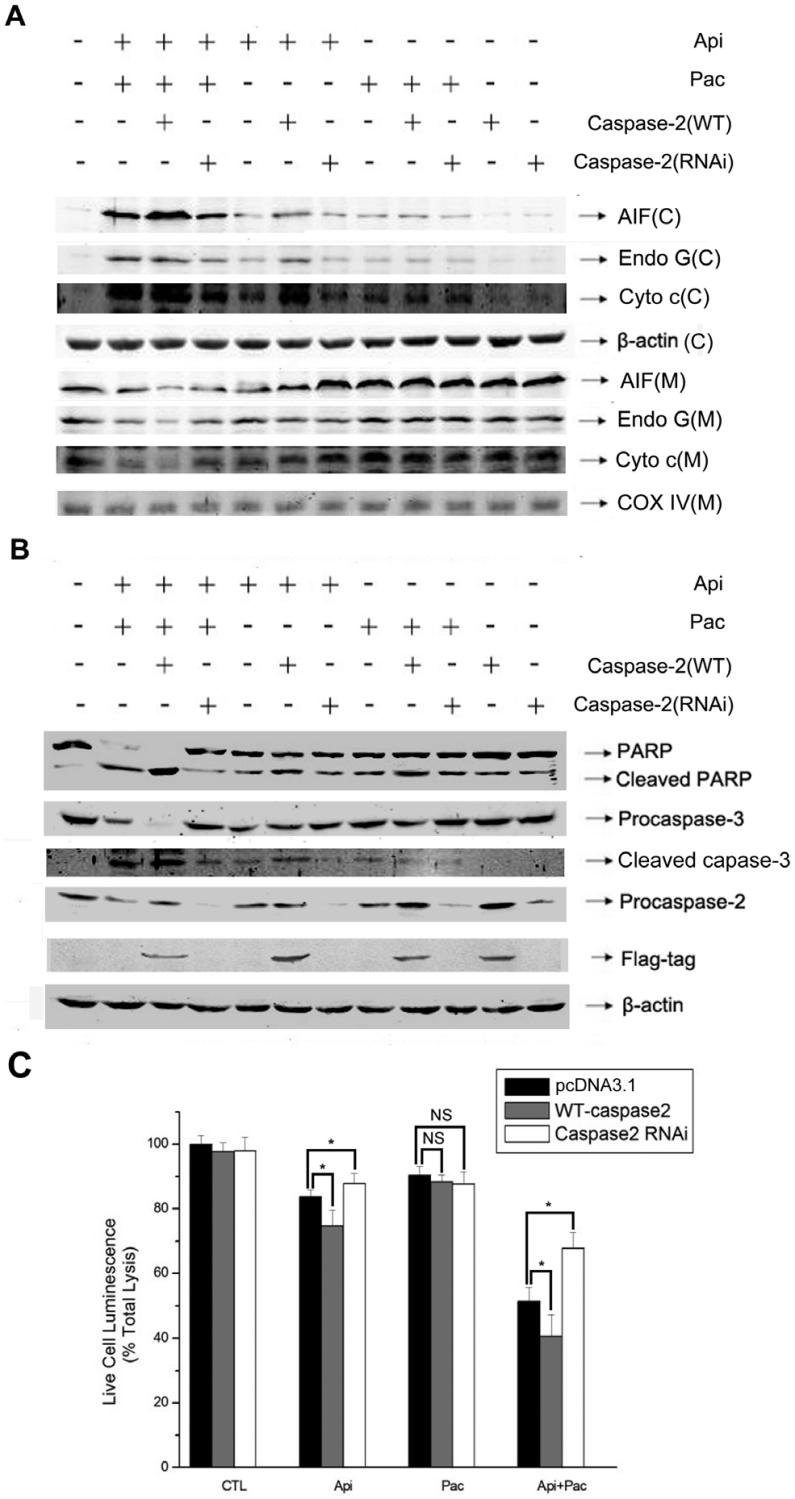
Caspase-2 cleavage is important for apigenin/paclitaxel-induced apoptosis. A, HeLa cells were transfected with flag-caspase2 or RNAi-caspase2. Forty-eight hours after transfection, cells were treated with apigenin (15 µM), paclitaxel (4 nM) or both of them for 24 hours. The mitochondrial fraction was separated from cytosolic fraction. The protein levels of AIF, Endo G and cytochrome c in both fractions were measured. β-actin and COX-IV were probed as the cytosolic and mitochondrial control respectively. Cytosolic and mitochondrial fractions were displayed as C and M respectively. B, Cells were treated as same as described in A. Protein levels of PARP, cleaved PARP, procaspase-2 and procaspase-3 were determined by Western blot analysis. C, Flag-caspase2 or RNAi-caspase2 was transfected to HeLa cells, and 48 hours after tranfection, cells were treated with apigenin (15 µM), paclitaxel (4 nM) or both of them for 24 hours. Cell vitality was measured with a cytotoxicity detection kit from Promega. The experiment was independently repeated for three times and data were shown as mean±S.D. *p<0.05 compared with empty-plasmid transfected group.

To confirm the importance of caspase-2 in apigenin/paclitaxel-triggered mitochondrial membrane permeabilization and apoptosis, we perform caspase-2 over-expression and knock-down experiments and measured AIF, Endo G and cytochrome c releasing from mitochondrion in HeLa cells. Over-expression of caspase-2 significantly enhanced apigenin or apigenin/paclitaxel-induced increase of AIF, Endo G and cytochrome c in cytoplasma fraction and decrease of AIF, Endo G and cytochrome c in mitochondrial fraction (Fig. 6A). As expected, caspase-2 knock-down resulted in the opposite on apigenin or apigenin/paclitaxel induced changes of AIF, Endo G and cytochrome c in HeLa cells (Fig. 6A). Furthermore, as shown in Fig. 6B, over-expression of caspase-2 apparently increased apigenin/paclitaxel-induced cleavage of caspase-3 and PARP and knock-down of caspase-2 inhibited such effect of apigenin/paclitaxel. We next detected cell vitality to evaluate mediated effect of caspase-2 on apigenin/paclitaxel triggered HeLa cell apoptosis. Over-expression of caspase-2 caused about 10% decrease of cell vitality both in apigenin and co-treatment groups, and caspase-2 silence significantly increased cell vitality. However, there was no significant change in paclitaxel treated groups no matter whether caspase-2 was over-expressed or down-expressed. Combined, the above data suggested that caspase-2 did play an important role in apigenin/paclitaxel induced apoptosis of HeLa cells.

## Discussion

It has been well recognized that apigenin and many other flavonoids act as anti-oxidants. Their ability to scavenge ROS was believed to be related with their effects on anti-cancer, chemoprevention [41] and anti-inflammation [42]. However, up until now, only a few attentions have been paid to the pro-oxidant effect of apigenin [22]. The reason for why flavonoids possess both pro-oxidant and anti-oxidant capability is unclear. Some studies suggested that it depended on the dose of flavonoids and the type of cells [24]. Cancer cells are frequently under persistent oxidative stress because of oncogenic stimulation, increased metabolic activity, and mitochondrial malfunction [43], [44].

In the present study, we demonstrated that apigenin potentiated ROS accumulation in HeLa cells, which correlated closely with its inhibitory effect on SOD activity. SODs are the only enzymes dismuting superoxide radicals. We noticed that Zn^2+^, Cu^2+^ and Mn^2+^ could reverse apigenin-induced suppression of SOD activity whereas these metal ions did not affect the basal SOD activity. It was probably that apigenin formed a stable complex with metal ions and acted as a competitive inhibitor of SOD. More detailed studies are needed in the future. In fact, two stable coordination geometries of the aluminum complex of apigenin have been identified [34].

Our study indicated that apigenin/paclitaxel-induced the cleavage of caspase-2 in a ROS dependent manner and consequently led to MMP which resulted in leakage of pro-apoptotic proteins from mitochondrion, such as SMAC/DIABLO [45], [46], Htr2A/Omi [47] and cytochrome c [46]. These data was consistent with previous studies in neuron stem cells [48], leukemia cells [49], and MCF-7 cells (Fig. S1), which suggested ROS as a precipitating factor in caspase-2 cleavage in these cell lines. Numerous *in vivo* studies have showed that SOD could highly express in aggressive human solid tumors [50]. It has also been reported that MnSOD can be induced by cytotoxic drugs and this may be related with development of drug resistance and a poor prognosis after chemotherapy in cancer cells [51], [52]. In our current study, apigenin treatment caused the suppression of SOD, particularly the suppression of MnSOD. On the other hand, DETC, a well approved inhibitor of both CuZnSOD and MnSOD enhanced paclitaxel induced apoptosis. These results strongly suggested that SOD activity was a critical factor in paclitaxel induced apoptosis and apigenin enhanced paclitaxel-induced apoptosis through suppressing SOD activity.

Though it have been addressed that apigenin was a potential inducer of intracellular oxidative stress, and apigenin-induced production of ROS play a vital role in apoptosis signal [22], very limited data was presented on setting apigenin as a candidate to be used in combination with traditional anti-neoplastic agents. Paclitaxel, a potent traditional anti-cancer drug, can cause both mitotic arrest and apoptotic cell death [53]. However, a high dose of paclitaxel applied clinically shows serious side effects and drug resistance [1]–[5]. Our results demonstrated the synergistic effects of apigenin and paclitaxel on promoting HeLa cell apoptosis. Apigenin sensitized HeLa cells to paclitaxel-induced apoptosis through suppressing MnSOD and cooperated with paclitaxel to stimulate caspase-2 activation. The results that AIF, EndoG and cytochrome c were releasing from mitochondrion indicates both caspase dependent and caspase independent machineries are working in the apigenin enhanced paclitaxel induced apoptosis. Apigenin, as a potent natural anti-cancer compound, showed little cytotoxicity toward non-transformed cells and possessed non-mutagenic nature. The combination use of apigenin and paclitaxel will greatly improve the efficiency of paclitaxel as a chemotherapeutic drug. Reduction of the dose of paclitaxel in cancer therapy will decrease its adverse reactions.

In conclusion, our study demonstrates that combination of apigenin and paclitaxel possesses a more apparent anti-cancer activity *in vitro* compared with each of them alone. Mechanically, apigenin suppresses the activity of SOD to lead cancer cells sensitive to cytotoxity of paclitaxel. Furthermore, ROS-induced activation of caspase-2 and depolarization of MMP participate in apigenin and paclitaxel induced cancer cell apoptosis. Therefore, this study indicates the synergistic anti-cancer effects of apigenin and paclitaxel in tumor chemotherapy.

## Supporting Information

Figure S1 Dot plots of annexin V/PI staining on viable versus apoptotic of a drug resistant cell line MCF-7. Cells were either left untreated or as previously described with 15 µM apigenin and 4 nM paclitaxel. At the indicated time of 24 hours, cells were stained for annexin V/PI. Analyses were conducted on 5,000 cells in each case.
(TIF)
