# Supplementary figures and images for: Synergistic Effects of Apigenin and Paclitaxel on Apoptosis of Cancer Cells
Source: PLoS One. 2011 Dec 21;6(12):e29169. doi: 10.1371/journal.pone.0029169 (PMC3244456; doi:10.1371/journal.pone.0029169)

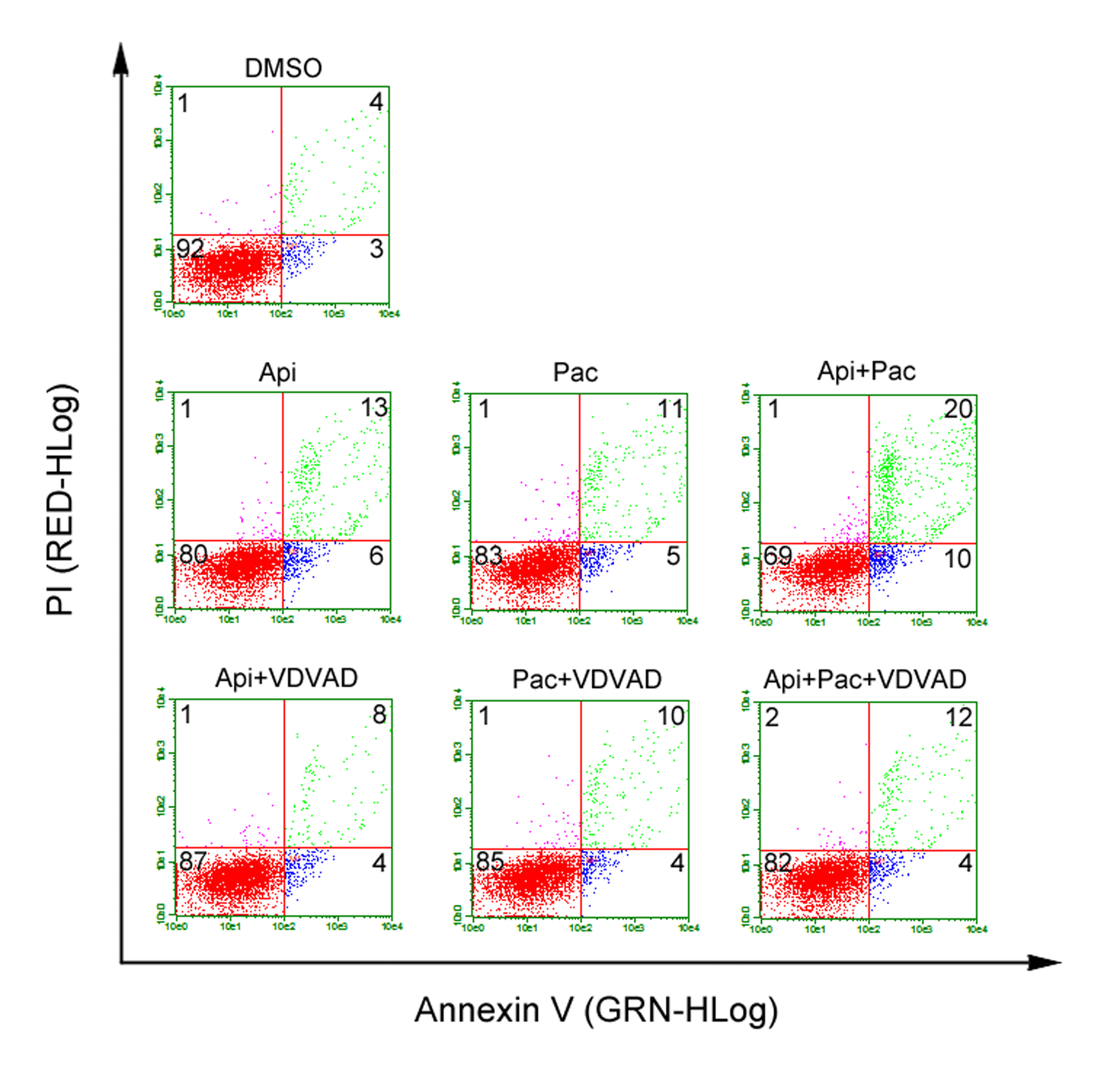

Supplement: Figure S1 — Dot plots of annexin V/PI staining on viable versus apoptotic of a drug resistant cell line MCF-7. Cells were either left untreated or as previously described with 15 µM apigenin and 4 nM paclitaxel. At the indicated time of 24 hours, cells were stained for annexin V/PI. Analyses were conducted on 5,000 cells in each case. (TIF) [file pone.0029169.s001.tif]
